# Multiple Subcutaneous Metastases in Endometrial Cancer Treated With Systemic Chemotherapy: A Case Report and Review of the Literature

**DOI:** 10.7759/cureus.76924

**Published:** 2025-01-04

**Authors:** Ryo Higashide, Seiichi Endo, Mayu Akita, Nanami Kawakami, Koji Shimabukuro

**Affiliations:** 1 Department of Obstetrics and Gynecology, Tsuchiura Kyodo General Hospital, Ibaraki, JPN; 2 Department of Obstetrics and Gynecology, JA Toride Medical Center, Ibaraki, JPN

**Keywords:** clear cell carcinoma, endometrial cancer (ec), endometrioid carcinoma, lenvatinib-induced hepatic encephalitis, msi-high, subcutaneous metastasis, tmb-high

## Abstract

Subcutaneous metastasis of endometrial cancer (EC) is an exceedingly rare phenomenon, and the mechanisms underlying its pathogenesis remain insufficiently elucidated. We report the case of a 69-year-old multiparous woman diagnosed with stage IB EC, histologically classified as grade 3 endometrioid and clear cell carcinoma. Approximately one month following primary surgical intervention, the patient developed subcutaneous masses on her back, axilla, and buttock. Histopathological evaluation, in conjunction with PET-CT, confirmed a systemic recurrence of EC. She was subsequently treated with a combination of paclitaxel and carboplatin, with a remarkable therapeutic response; after six cycles of chemotherapy, nearly all metastatic sites exhibited complete resolution. She was then transitioned to a regimen of lenvatinib and pembrolizumab. However, one week after the initiation of this treatment, she developed hepatic encephalopathy, which was presumed to be lenvatinib-induced, and was successfully managed with aminoleban injections, resulting in a full recovery of consciousness. Follow-up diagnostic imaging confirmed a complete response to therapy. The patient is currently receiving pembrolizumab as maintenance therapy and has remained recurrence-free for 18 months. While no standardized therapeutic protocol exists for the management of subcutaneous metastasis in EC due to its extremely low incidence, this report provides evidence for the potential efficacy of chemotherapy combined with targeted immunotherapy in the treatment of unresectable subcutaneous metastases in EC.

## Introduction

Endometrial cancer (EC) is the most prevalent gynecological malignancy in Japan. While early-stage EC typically demonstrates a favorable prognosis, advanced and recurrent cases pose significant therapeutic challenges, particularly in achieving complete cancer resection and curative outcomes. Despite a five-year survival rate of 95.7% for uterine localized EC, the survival rates for patients with lymph node metastasis and distal metastasis are considerably lower, at 73.2% and 20.1%, respectively, in Japan [1]. Chemotherapeutic advances for EC have led to the development of multiple treatment regimens; however, in many instances, these therapies fail to yield effective results in advanced or recurrent EC cases.

Subcutaneous metastasis of EC is exceedingly rare, with only a few cases reported so far, and the underlying mechanisms remain poorly understood [2-6]. EC metastasizing to soft tissues often signifies extensive systemic spread, contributing to a poor prognosis. There are currently no reports documenting the successful treatment of subcutaneous metastasis in EC. We discuss a case of subcutaneous metastasis occurring one month after initial surgery in a patient with EC, which was effectively managed through systemic chemotherapy.

## Case presentation

**Figure 1:**
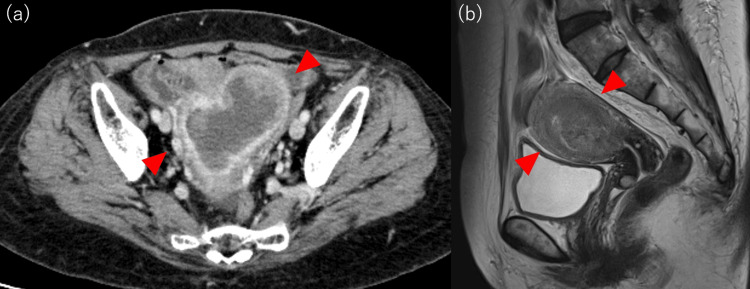
Preoperative imaging (a) A contrast-enhanced CT scan of the pelvis reveals a mass localized to the uterus, with no evidence of metastatic involvement (red arrowhead). (b) A T2-weighted MRI of the pelvis demonstrates a tumor confined to the uterine corpus, with an estimated invasion extending beyond 50% of the myometrium (red arrowhead) CT: computed tomography; MRI: magnetic resonance imaging

A 69-year-old multiparous woman presented to a tertiary hospital with a year-long history of vaginal bleeding. A transvaginal ultrasound revealed a solid tumor within the uterine cavity, while a CT scan showed no signs of metastasis. MRI indicated that the tumor was confined to the uterine corpus, with an estimated invasion of more than 50% of the myometrium (Figure 1).

An endometrial biopsy confirmed the diagnosis of EC. The patient subsequently underwent an abdominal total hysterectomy, bilateral salpingo-oophorectomy, partial omentectomy, and pelvic lymphadenectomy. Histopathological analysis identified endometrioid carcinoma with a clear cell component, grade 3, classified as pT1b, pN0, corresponding to stage IB according to the International Federation of Gynecology and Obstetrics (FIGO) 2009 classification. The tumor was confined to the uterine corpus, with no evidence of metastasis. There was no evidence of lymphatic invasion; however, venous invasion was identified. Cancer genomic profiling revealed a high microsatellite instability (MSI-High) status and a tumor mutation burden (TMB) of 52 mutations per megabase (Mb). The patient was scheduled for adjuvant chemotherapy.

**Figure 2:**
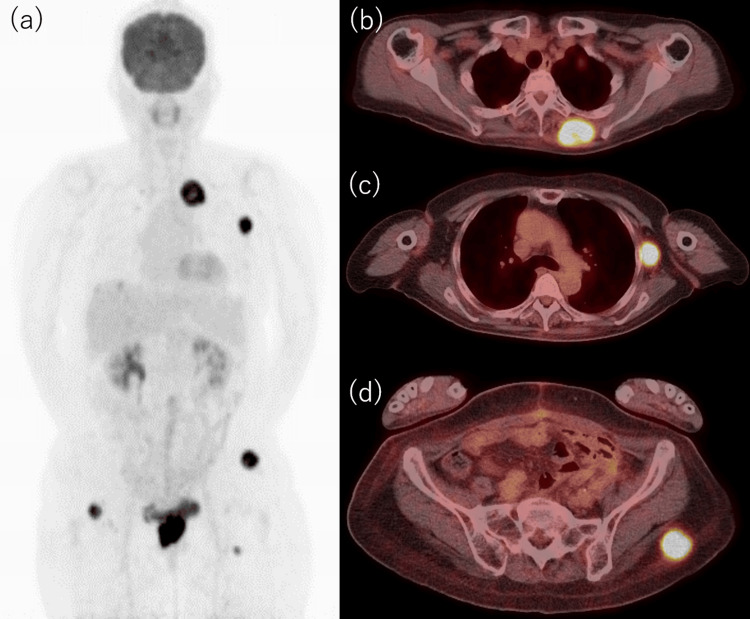
Postoperative PET-CT imaging one month after primary surgery (a) FDG uptake is observed diffusely throughout the body. (b) FDG uptake is noted in the left posterior thoracic region. (c) FDG uptake is identified in the left axillary region. (d) FDG uptake is seen in the left buttock region. All subcutaneous metastases were palpable upon physical examination FDG: 18F-fluorodeoxyglucose; PET-CT: positron emission tomography-computed tomography

One month post-surgery, before the initiation of adjuvant chemotherapy, the patient experienced recurrent vaginal bleeding. A gynecological examination identified a 2-cm bleeding mass in the vagina, and histological analysis confirmed a recurrence of EC. In addition to the vaginal mass, the patient also noted subcutaneous masses on her back, axilla, and buttock. A PET-CT scan revealed 18F-fluorodeoxyglucose (FDG) uptake in the lungs, liver, multiple subcutaneous regions, and pelvic floor (Figure 2).

**Figure 3:**
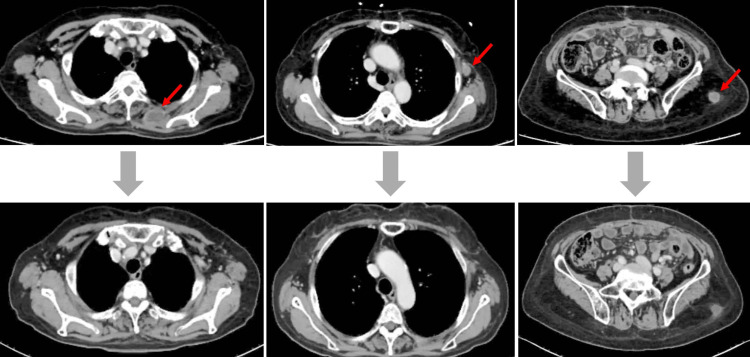
CT images showing response to chemotherapy The upper row displays pre-treatment images, while the lower row presents post-treatment images following three cycles of paclitaxel and carboplatin. A nearly complete resolution of all metastatic lesions is observed. Red arrows indicate subcutaneous metastases CT: computed tomography

The patient was diagnosed with a systemic recurrence of EC and was subsequently planned for chemotherapy with paclitaxel and carboplatin. Following one cycle of chemotherapy, the size of the subcutaneous masses was reduced. After six cycles of paclitaxel and carboplatin, a CT scan demonstrated an almost complete response to all metastatic lesions (Figure 3).

Upon completion of chemotherapy, the patient was transitioned to treatment with lenvatinib and pembrolizumab as part of an ongoing treatment regimen. One week after the initiation of lenvatinib and pembrolizumab, the patient presented to the emergency department with impaired consciousness [Glasgow Coma Scale (GCS) of E4V4M5]. Laboratory tests revealed elevated serum ammonium levels and triphasic waves were detected on an electroencephalogram. Cranial CT and MRI scans showed no significant abnormalities, and the patient was diagnosed with hepatic encephalopathy. She responded rapidly to aminoleban injections, regaining full alertness and orientation the following day. After a seven-day hospitalization, she was discharged without sequelae. A follow-up CT scan revealed a complete response with no residual tumor. The patient is currently receiving pembrolizumab as maintenance therapy and has remained recurrence-free for 18 months with no severe adverse events.

## Discussion

EC is the most prevalent gynecological malignancy worldwide. In Japan, more than 17,000 women were diagnosed with EC, and nearly 3,000 died in 2020, according to the cancer statistics provided by the National Cancer Center and the Ministry of Health, Labor and Welfare of Japan. The incidence of EC continues to rise annually, with over 400,000 new cases reported globally in 2020 [7]. Historically, treatment strategies following primary surgery for EC were largely based on uterine histopathological features, including tumor grade, depth and extent of invasion, and the presence of lymph node metastasis, which were considered key prognostic factors. Based on these factors, gynecologists classified recurrence risk into high, intermediate, and low categories to guide the decision to administer adjuvant chemotherapy.

In recent years, new risk stratification models have been introduced, and treatment approaches for EC have increasingly incorporated molecular classifications [8]. Current clinical practice emphasizes evaluating histopathological status in conjunction with molecular markers such as mismatch repair (MMR) status, POLE mutations, and TP53 mutations to assess prognosis more accurately. Furthermore, the advent of new therapeutic regimens has made the treatment of EC more complex. Notable advancements include the introduction of pembrolizumab for MSI-High cancers [9], the combination of lenvatinib and pembrolizumab [10], as well as the use of dostarlimab [11], and the combination of durvalumab and olaparib [12], which have demonstrated efficacy in treating EC.

In the case presented, we initially anticipated a poor prognosis due to the rapid and diffuse progression of the tumor. However, contrary to expectations, the patient achieved a complete response and remains tumor-free. This outcome underscores the potential effectiveness of systemic chemotherapy in the management of EC, particularly in cases with aggressive disease characteristics.

To the best of our knowledge, only a few cases of subcutaneous metastasis in EC have been reported in the literature (Table 1) [2-6].

**Table 1:** Literature review of subcutaneous metastasis in endometrial cancer N/A: not applicable

Author	Patient age, years	Stage	Histology	Primary treatment	Adjuvant therapy	Duration from primary surgery to subcutaneous metastasis	Location of subcutaneous metastasis	Treatment for subcutaneous metastasis	Results
La Fianza et al. [2]	67	IA	Clear cell carcinoma	Colpohysterectomy	40 Gy of external radiation	Eight months	Right abdominal wall	Surgical removal	Progressive disease
Dikmen et al. [3]	62	IA	Endometrioid and mucinous, grade 2	Extra fascial hysterectomy plus bilateral salpingo-oophorectomy	None	16 months	Anterior abdominal wall	Surgical removal and local radiotherapy	N/A
M'rabet et al. [4]	72	IC	Endometrioid carcinoma, grade 1	Total hysterectomy, bilateral salpingo-oophorectomy, pelvic and para-aortic lymph node sampling	None	Six months	Trunk, extremities, scalp	None	Death after two weeks from recurrence
Bacalbasa et al. [5]	66	IIIA	Moderately differentiated endometrioid carcinoma	Total hysterectomy, bilateral salpingo-oophorectomy, pelvic and para-aortic lymphadenectomy	Nine cycles of sindaxel and Carboplatin	18 months	Anterior abdominal wall	Surgical removal	N/A
Shurie et al. [6]	45	IIA	Endometrioid carcinoma, grade 3	Radical hysterectomy	Six cycles of carboplatin and paclitaxel	Immediately after adjuvant chemotherapy	Right leg	Pegylated liposomal doxorubicin and local radiation	Death after a year from recurrence
Current study	69	IB	Endometrioid and clear cell carcinoma, grade 3	Total hysterectomy, bilateral salpingo-oophorectomy, partial omentectomy, pelvic lymphadenectomy	None	One month	Back, axilla, breech, lungs, liver, pelvic floor	Paclitaxel and carboplatin, lenvatinib and pembrolizumab	Complete response

Fianza et al. described a case of subcutaneous metastasis to the right abdominal wall eight months after primary surgery for stage IA EC, with histological analysis revealing clear cell carcinoma [2]. Although the tumor was surgically resected, recurrence was seen in the pelvic lymph nodes and abdominal wall two months later, with no further effective treatment options available. Dikmen et al. reported a case of metastasis to the anterior abdominal wall 16 months following primary surgery for stage IA EC, with a mixed histology of endometrioid and mucinous carcinoma [3]. Surgical resection was performed, followed by radiotherapy. M’rabet et al. described a case of diffuse subcutaneous metastasis involving the trunk and extremities six months after primary surgery for stage IC EC [4]. At the time of detection of the subcutaneous metastasis, the patient's performance status had significantly deteriorated, and no treatment was initiated. The disease progression was rapid and fatal, and the patient died within two weeks. Bacalbasa et al. documented a case of subcutaneous metastasis to the abdominal anterior wall 18 months after surgery for stage IIIA EC, with metastasis confined to the abdominal wall and removed surgically [5]. Shurie et al. presented a case of stage IIA EC where subcutaneous recurrence was found in the right leg after a radical hysterectomy followed by six cycles of chemotherapy [6]. Despite treatment with pegylated liposomal doxorubicin and local radiation, the patient succumbed to metastatic EC one year after the development of subcutaneous metastasis.

Treatment approaches for subcutaneous metastasis in EC varied across these cases. In cases of oligometastatic disease, surgical resection may be a viable option. However, in the present case, given the widespread nature of recurrence, involving multiple regions of the body, we decided against surgical intervention and initiated systemic chemotherapy instead. Cutaneous metastasis is also a rare phenomenon, though several case reports and reviews have documented its occurrence [13]. Breast cancer is the most common primary source of cutaneous metastasis, followed by cancers of the colon, melanoma, and the ovary [14]. It is generally believed that cutaneous metastasis occurs through both hematogenous and lymphatic routes. In EC, metastasis to the umbilicus, known as a Sister Mary Joseph nodule, is the most frequently observed pattern of cutaneous involvement, although the overall incidence of cutaneous metastasis in EC remains rare [2]. There is no established treatment protocol for skin metastasis, and it typically has a poor prognosis, as the disease will have often disseminated extensively throughout the body by the time of detection. Hence, gynecologists should remain vigilant for the possibility of cutaneous metastasis during the follow-up of patients with gynecologic cancers.

In the case presented, the combination of conventional chemotherapy and pembrolizumab demonstrated significant therapeutic efficacy. The patient's cancer genomic profile revealed MSI-High and TMB-High, suggesting that immune checkpoint inhibitors may have played a crucial role in the treatment response. Previous studies have shown that approximately 33% of endometrioid EC exhibit MSI-High status, with this feature being most frequently observed in grade 3 tumors [15]. High-grade tumors generally exhibit increased susceptibility to immune-based therapies compared to low-grade tumors. Given the higher prevalence of MSI-High in EC relative to other malignancies, genomic profiling of tumor status in EC is a valuable tool for guiding treatment decisions. By promptly assessing the genomic characteristics of the tumor, we were able to anticipate and customize the treatment approach at the time of recurrence, optimizing therapeutic outcomes.

Lenvatinib-induced hepatic encephalopathy is a rare complication, and there are no prior reports of its occurrence in patients with EC treated with lenvatinib [10]. Lenvatinib has been utilized for the treatment of unresectable hepatocellular carcinoma since 2018 and has demonstrated significantly superior efficacy compared to the previous standard chemotherapy regimen, sorafenib [16]. Among a cohort of 476 patients treated with lenvatinib, 18 cases (3.8%) of lenvatinib-induced hepatic encephalopathy were reported. This incidence increased to 7.4% when only the Japanese subset of the study population was considered [17,18]. The median onset time of hepatic encephalopathy was 16.5 days after initiating lenvatinib therapy.

The pathophysiology of hepatic encephalopathy is primarily attributed to hepatic failure or the development of a portosystemic shunt, which leads to the accumulation of toxins, particularly ammonia, and subsequent cerebral dysfunction [19]. In a case report by Namba et al., occlusion of a portosystemic shunt after lenvatinib-induced hepatic encephalopathy enabled the patient to resume lenvatinib treatment without recurrence of encephalopathy [20]. When a collateral venous pathway is present, ammonia produced in the gastrointestinal tract bypasses the liver, resulting in elevated blood ammonia levels and the onset of hepatic encephalopathy. In the case presented, a collateral vein originating from the splenic vein and inferior mesenteric vein, which drained into the inferior vena cava, was identified on the pre-surgery CT scan. This anatomical feature may have contributed to the development of elevated ammonia levels and the onset of hepatic encephalopathy in our patient.

## Conclusions

Subcutaneous metastasis of EC is an exceedingly rare entity and is scarcely documented in the current medical literature. Given the rarity of such occurrences, a poor prognosis was initially anticipated in our patient. However, the patient exhibited a remarkable response to systemic chemotherapy, resulting in the complete resolution of the tumor. This report underscores the potential of systemic chemotherapy as an effective therapeutic strategy for managing unresectable recurrent EC, suggesting its potential role in achieving durable remission in such rare and challenging clinical presentations.
